# Case Report: Rapid response to gemtuzumab-ozogamicin in a pediatric patient with refractory systemic mastocytosis with AML1::ETO+ acute myeloid leukemia

**DOI:** 10.3389/fimmu.2025.1566805

**Published:** 2025-04-09

**Authors:** Song Xue, Man Chen, Hui-Peng Sun, Tong Wang, Li-Na Zhang, Xing-Yu Cao

**Affiliations:** ^1^ Department of Bone Marrow Transplant, Beijing Lu Daopei Hospital, Beijing, China; ^2^ Division of Pathology & Laboratory Medicine, Beijing Lu Daopei Hospital, Beijing, China; ^3^ Department of Laboratory Medicine, Hebei Yanda Lu Daopei Hospital, Langfang, China; ^4^ Department of Bone Marrow Transplant, Hebei Yanda Lu Daopei Hospital, Langfang, China

**Keywords:** gemtuzumab-ozogamicin., systemic mastocytosis (SM), AML1::ETO, acute myeloid leukemia, systemic mastocytosis with an associated hematological neoplasm

## Abstract

This article describes a 6-year-old patient diagnosed with “systemic mastocytosis with AML1::ETO+ AML”, he experience refractory disease during the course of treatment and salvage treatment was ineffective. The patient was administered gemtuzumab-ozogamicin therapy, resulting in rapid remission.

## Introduction

Systemic mastocytosis (SM) is a neoplasm characterized by the proliferation of mast cells (MCs) in extracutaneous organs. SM encompasses various entities, each with distinct clinical manifestations and prognostic outcomes (1). In the Mayo series, SM with associated hematologic neoplasm (SM-AHN) was the second most prevalent subgroup of SM, accounting for 40% of the cases (2). AHNs encompass a wide range of hematologic malignancies, including overlapping syndromes of MDS/MPN (such as CMML, which is the most common), as well as MDS, AML, and, less frequently, lymphomas and plasma cell neoplasms (3). KIT mutations are associated with enhanced proliferation of MCs and are frequently detectable in AML1::ETO+ AML, AML that occurs concomitantly often harbors the same KIT mutations to those found in neoplastic MCs, existing evidence suggests that leukemic blasts and MCs derive from a common malignant progenitor (4, 5). The prognosis for SM with AML1::ETO+ AML is extremely poor, posing significant therapeutic challenges (5–7). Aberrant MCs in SM display a distinct immune phenotype and express myeloid-associated antigens, indicating the potential feasibility of targeted therapy directed against specific cell surface markers. In this study, we present a case of a pediatric patient diagnosed with refractory SM with AML1::ETO+ AML, who experienced rapid remission after receiving gemtuzumab-ozogamicin (GO) therapy.

## Case presentation

In August 2024, a pediatric patient sought medical attention at a local hospital, presenting with generalized petechiae lasting for over 20 days. Complete blood count revealed a white blood cell count of 16.3×10^9/L, a hemoglobin level of 76 g/L, and a platelet count of 12×10^9/L. Bone marrow smear analysis revealed that 53% of the cells were myeloblasts. Flow cytometry (FCM) of the bone marrow identified 32.15% of abnormal cells expressingCD117, CD34, HLA-DR, CD123, partially expressing CD19, and weakly expressing CD33, CD13, CD38, and MPO. These cells did not express TDT, cCD3, cCD79a, or other myeloid and lymphoid markers, they were identified as abnormal myeloid blasts. Furthermore, genetic sequencing identified the presence of the AML1::ETO fusion gene along with KIT N822K (VAF 9.19%) and KIT W577R (VAF 33.81%) somatic mutations. Chromosomal analysis resulted in a karyotype of 46,XY,del(7)(q32),t(8;21)(q22;q22.1)[1]/46,idem,del(9)(q12q21)[14].Based on the obtained findings, the patient was definitively diagnosed with AML.

The patient initially received the MAH regimen (mitoxantrone, cytarabine, and homoharringtonine) as induction therapy. Subsequent bone marrow morphology examinations revealed no response to the treatment. Following administration of the EA regimen (etoposide and cytarabine), bone marrow aspiration showed no signs of leukemic remission. Subsequently, the patient underwent chemotherapy with the CLAG regimen (claribine, cytarabine, and G-CSF).Unfortunately, peripheral blood examination showed no achievement of remission. The patient was then referred to our hospital for advanced treatment, where a comprehensive evaluation of disease condition was conducted.

**Figure 1:**
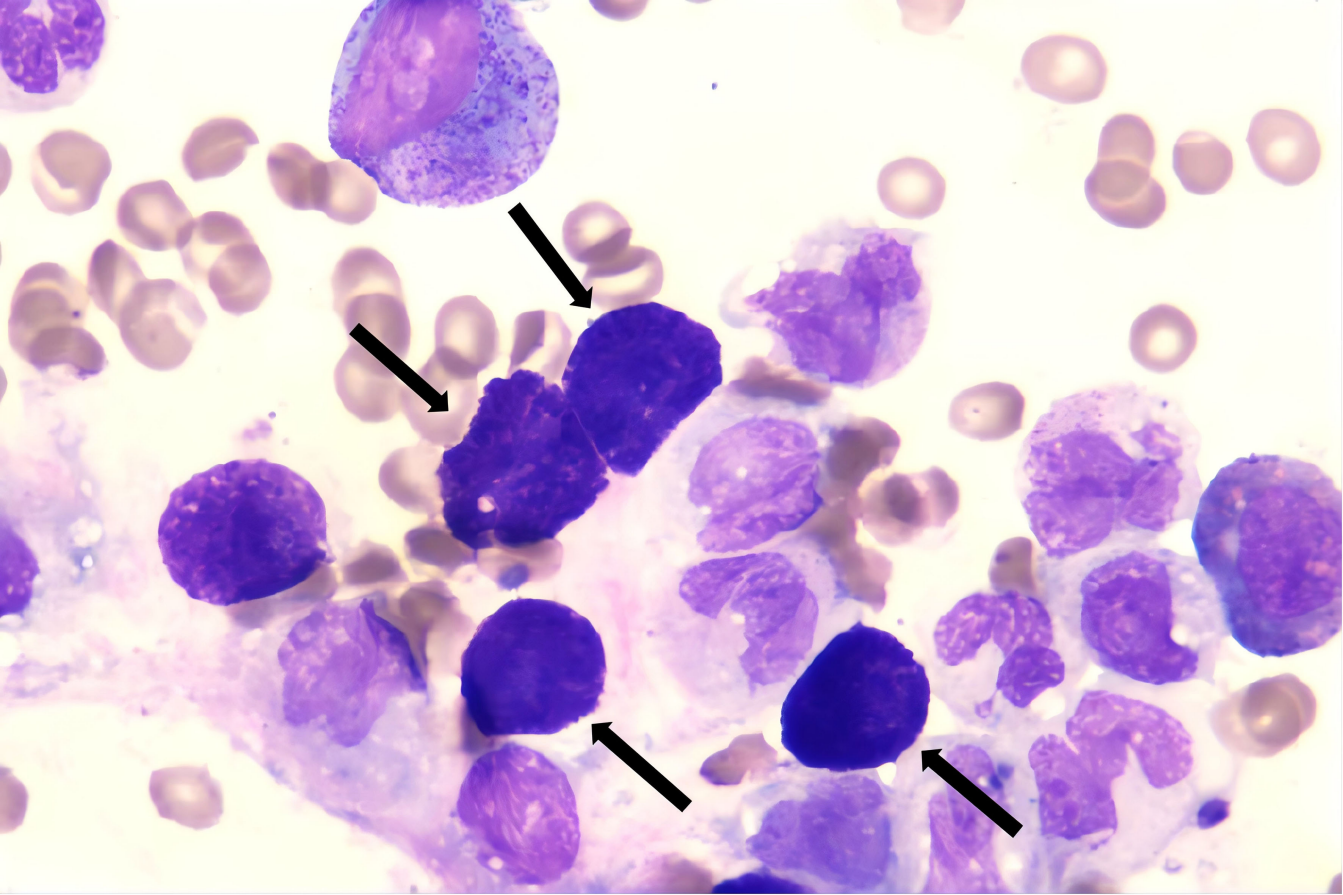
Mast cells (black arrow), present in clusters, are clearly visible on bone marrow smears. (Wright’s stain ×100).

**Figure 2:**
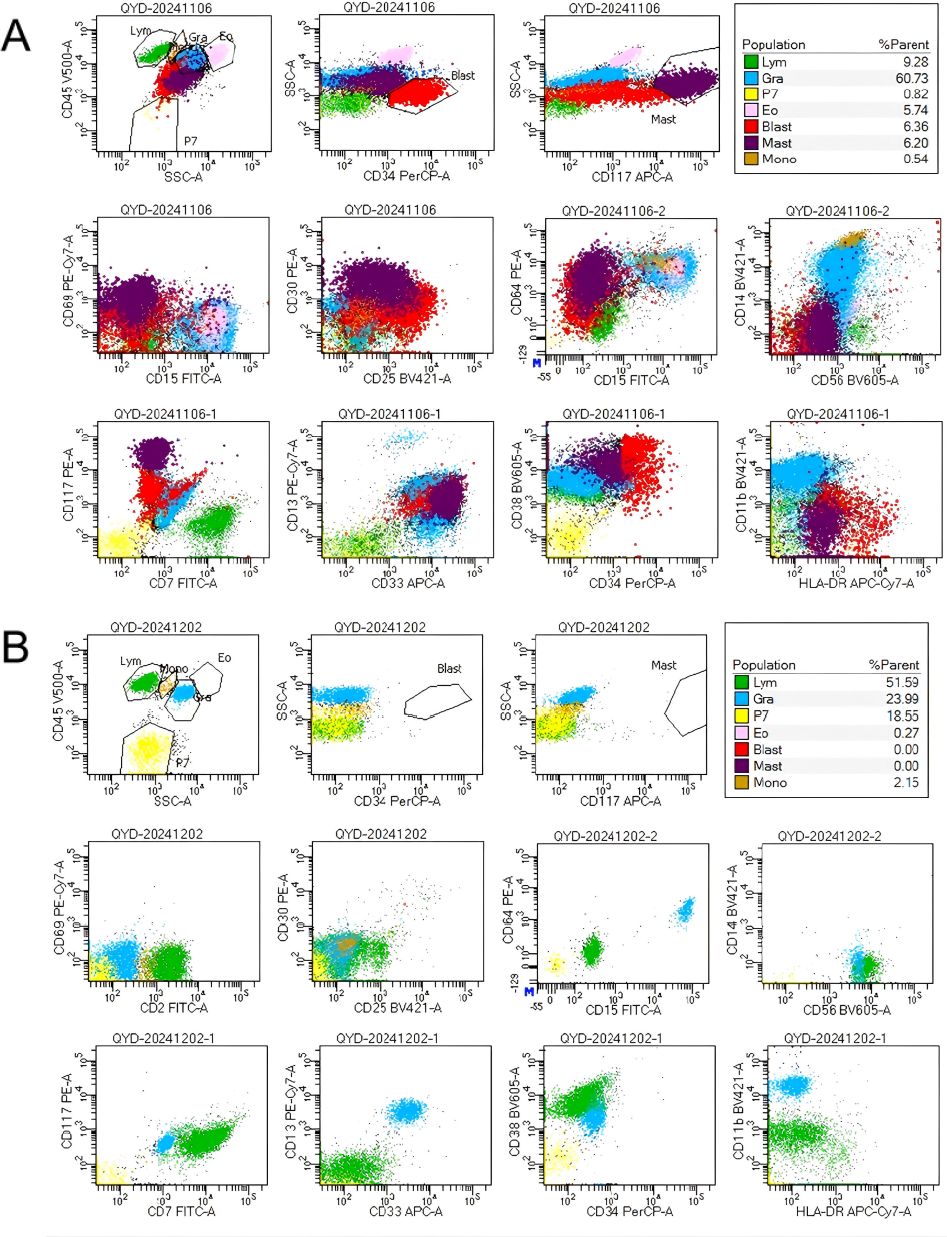
**(A)** Flow cytometry analysis of bone marrow identified two distinct abnormal cells: (1) Malignant immature myeloid cells, labeled as the ‘red group’, comprising 6.36% of nucleated cells, expressing CD34, CD117dim, CD13, CD33bri, CD38, HLA-DRdim, CD64dim, CD30, and CD25, and negative for CD7, CD11b, CD14, CD56, CD15, CD69.(2) Abnormal mast cells, designated the ‘purple group’, accounted for 6.2% of nucleated cells. These cells demonstrated increased SSC, enhanced CD117 and CD33 expression, decreased CD69 expression, abnormal CD64 and CD30 expression, and were negative for CD25, CD34, CD15. **(B)** After GO treatment, FCM of the bone marrow demonstrated the absence of leukemic cells and abnormal mast cells.

Despite the patient’s persistent and severe neutropenia, bone marrow smears showed that primitive granulocytes comprised 10% of total cells, whereas mast cells were markedly increased, comprising 12% of the cells and exhibiting abnormal morphologies. Notably, clusters of abnormal mast cells were observed (**Figure 1**). FCM analysis of bone marrow samples revealed that 6.36% of nucleated cells were positive for CD34, CD117dim, CD13, CD33bri, CD38, HLA-DRdim, CD64dim, CD30, and CD25, and negative for CD7, CD11b, CD14, CD56, CD15, CD69, and CD16.These cells were characterized as malignant immature myeloid cells (**Figure 2**). Abnormal mast cells (**Figure 2**), which comprised 6.2% of nucleated cells, demonstrated increased SSC, enhanced CD117 and CD33 expression, decreased CD69 expression, abnormal CD64 and CD30 expression, and were negative for CD25, CD34, CD15, and CD16. The AML1::ETO transcript level had surged to 281.736%. Following the laboratory test results, the patient’s diagnosis was updated to SM with AML1::ETO+ AML.

Due to the patient’s lack of response to three consecutive cycles of chemotherapy, further combination therapy was not pursued. The patient described herein possesses two mutations within the KIT gene: one in exon 11 and another in exon 17. Given that Avapritinib can inhibit mutations in both exons, it is an appropriate therapeutic option for this patient (8, 9). In real world practice, avapritinib demonstrates limited efficacy in treating KIT exon 11-mutated SM (10). Furthermore, considering the patient’s low white blood cell count, avapritinib was not administered to avert severe neutropenia and the risk of subsequent infections. Considering the robust expression of CD33 on both leukemic cells and abnormal mast cells in the patient, the administration of GO represents a logical therapeutic option. After thorough consultation and careful consideration, the patient’s parent provided both oral and written consent for the GO therapy. The patient subsequently received a weekly dosage of 3 mg/m²of GO. During treatment, only mild abdominal discomfort was observed, with no other treatment-related adverse reactions. Following two cycles of GO treatment, the patient underwent a bone marrow aspiration, revealing complete remission on the smear. FCM of the bone marrow demonstrated the absence of leukemic cells and abnormal mast cells, with a reduction in AML1::ETO gene quantitation to 0.372%. Given the patient’s favorable remission status, allogeneic hematopoietic stem cell transplantation (allo-HSCT) was promptly initiated.

**Figure 3:**
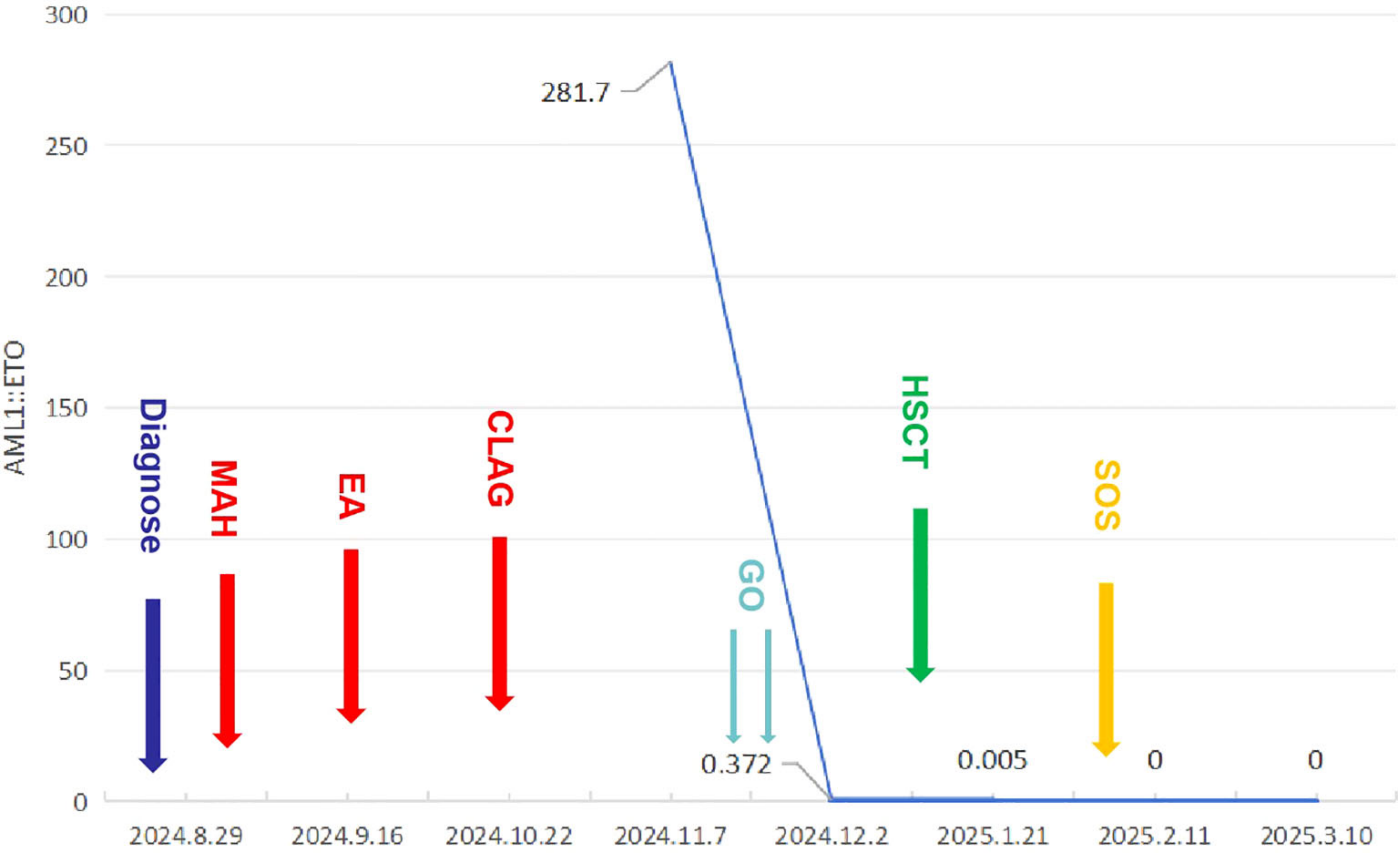
The patient’s complete treatment course, including variations in AML1::ETO transcript level throughout the therapeutic process.

On December 31, 2024, the patient underwent haploidentical allo-HSCT, neutrophils and platelets were implanted on days +12. At 3 weeks post-transplantation, the BM AML1::ETO transcript level was 0.002%. At 4 weeks post-transplantation, the patient exhibited abdominal distension and was subsequently diagnosed with sinusoidal obstruction syndrome (SOS). The condition improved after defibrotide treatment. At 6 weeks post-transplantation, the BM AML1::ETO transcript level was 0% (**Figure 3**).

Written informed consent for publication of this report and accompanying images was obtained from the parents.

## Discussion

AML with AML1-ETO fusion is generally associated with favorable outcomes due to high remission and survival rates. Approximately 25% of these cases harbor KIT mutations, which are linked to a poor prognosis (11, 12). A previous retrospective study demonstrated that 10% of AML1::ETO+ AML patients exhibited concurrent SM (13). A multicenter retrospective study, conducted in China from January 2009 to December 2022, identified 24 cases of SM with AML1::ETO+ AML across 16 centers and 212 cases of AML1::ETO+ AML with KIT mutations within the same timeframe (7). Based on the available data, we formulate a hypothesis that the coexistence of SM may contribute to some adverse effects of KIT on prognosis. Comprehensive screening is advisable for AML1::ETO+ AML patients with KIT mutations who demonstrate a suboptimal response to treatment, to ascertain the coexistence of SM. In cases of SM-AHN, abnormal mast cell infiltration can be masked by AHN cell infiltration. Consequently, we recommend repeating bone marrow aspiration and biopsy during the cytoreductive phase following chemotherapy, as this can improve the detection rate of SM, the rationale of this strategy is supported by our reported case.

SM with AML1::ETO+ AML often display a suboptimal response to standard induction chemotherapy, exhibiting frequent primary resistance to the treatment. Furthermore, aberrant MCs often persists even after achieving leukemia remission (6). The patient reported herein demonstrates primary drug resistance and failed to achieve remission despite undergoing salvage therapy with a cladribine-based regimen. Recently, small-molecule tyrosine kinase inhibitors (TKIs) targeting KIT have demonstrated promising clinical efficacy (1, 8, 14). Avapritinib has received FDA approval as a first-line treatment for adult patients with advanced SM, based on data from two clinical trials: EXPLORER (NCT02561988) (15) and PATHFINDER (NCT03580655) (16). However, considering the patient’s actual condition, avapritinib treatment is deemed unsuitable.

The abnormal mast cell immunophenotype is characterized by aberrant expression of CD25, CD2, or CD30. CD30, assessed using either flow cytometry or immunohistochemistry, is detected in 80% to 90% of SM cases (17). Therefore, targeting this antigen for the treatment of SM represents a viable strategy. A previous case series reported the clinical outcomes of four patients with CD30+ aggressive SM (ASM) or indolent SM who were treated with brentuximab vedotin (BV). Among the patients, two exhibited response. One patient demonstrated a durable response for over three years (18). In a subsequent Phase II clinical trial (NCT 01807598) (19), ten patients with CD30+ advanced SM, five of whom had SM-AHN, were enrolled. The results indicate that BV monotherapy is insufficient for the treatment of SM, especially in SM-AHN patients. Treating SM with AML1::ETO+ AML presents significant challenges, as it necessitates concurrent management of both disorders. In this scenario, CD30 may not serve as an optimal therapeutic target owing to its limited efficacy and the absence of expression on the surfaces of AML1::ETO+ AML cells. CD33 is expressed on early-committed myelomonocytic precursors and AML cells, but is absent on hematopoietic stem cells. Conversely, MCs exhibit high expression levels of CD33 on their surface membrane, irrespective of their maturation stage (20). Prior research has demonstrated that GO inhibits the proliferation and induces apoptosis in both normal and neoplastic MCs *in vitro*, GO neither induced secretion of histamine from MCs nor upregulated the anti-IgE-induced release of histamine in these cells (21). Presently, only one case report exist on the application of GO in the management of SM. Alvarez-Twose et al (22) reported a case of refractory mast cell leukemia negative for KIT mutations, which was successfully treated with monotherapy using GO, achieving sustained complete remission. Our successful case study, coupled with pertinent literature, has facilitated the progression of future clinical trials involving GO for the treatment of SM-AHN. Despite inherent limitations that may compromise the robustness of its conclusions, this case report still warrants recommending the therapeutic approach. The aim is to explore its potential benefits in patients with a poor prognosis.

Allo-HSCT significantly enhances the prognosis of patients with SM with AML1::ETO+ AML, warranting its strong recommendation for this patient population (7, 23). The reported patient achieved immunological remission and subsequently underwent allo-HSCT. Exposure to GO prior to transplantation was associated with an increased risk of developing specific transplant-related complications, notably SOS. Consequently, the implementation of more effective preventive measures for SOS is recommended. Allo-HSCT yields promising results in treating SM-AHN, with a 3-year overall survival rate of 74% (24); however, post-transplantation efficacy remains suboptimal for SM with AML1::ETO+ AML patients. The persistence of MCs in patients undergoing allo-HSCT constitutes a fascinating clinical observation, as post-transplantation relapse frequently leads to treatment failure (6).

A recent retrospective study has shown that the progression-free survival (PFS) following allo-HSCT for SM-AML is merely 0.7 years (25).The absence of a KIT D816V mutation, the presence of a complex karyotype, and the lack of pre-transplant TKI usage adversely affected PFS post allo-HSCT. Considering the patient’s currently favorable remission state, yet due to the presence of aforementioned risk factors, there is a high risk of disease relapse. Hence, initiating appropriate post-transplant maintenance therapy, such as TKIs, is a reasonable choice for treatment.

## Data Availability

The original contributions presented in the study are included in the article/supplementary material. Further inquiries can be directed to the corresponding author.
